# Quantitative Trait Loci Mapping Analysis for Cold Tolerance Under Cold Stress and Brassinosteroid-Combined Cold Treatment at Germination and Bud Burst Stages in Rice

**DOI:** 10.3389/fpls.2022.938339

**Published:** 2022-07-18

**Authors:** Zhifu Guo, Haotian Wang, Jialu Yao, Yishan Cheng, Wenzhong Zhang, Zhengjin Xu, Maomao Li, Jing Huang, Minghui Zhao

**Affiliations:** ^1^Key Laboratory of Agricultural Biotechnology of Liaoning Province, College of Biosciences and Biotechnology, Shenyang Agricultural University, Shenyang, China; ^2^Rice Research Institute, College of Agronomy, Shenyang Agricultural University, Shenyang, China; ^3^Rice Research Institute, Jiangxi Academy of Agricultural Sciences, Nanchang, China; ^4^Department of Agronomy, College of Agriculture, Purdue University, West Lafayette, IN, United States

**Keywords:** wild rice, QTL mapping, cold tolerance, brassinosteroids, germination and bud burst stages

## Abstract

Low temperature is one of the major abiotic stresses limiting seed germination and early seedling growth in rice. Brassinosteroid (BR) application can improve cold tolerance in rice. However, the regulatory relationship between cold tolerance and BR in rice remains undefined. Here, we constructed a population of 140 backcross recombinant inbred lines (BRILs) derived from a cross between a wild rice (Dongxiang wild rice, DXWR) and a super rice (SN265). The low-temperature germination rate (LTG), survival rate (SR), plant height (PH), and first leaf length (FLL) were used as indices for assessing cold tolerance under cold stress and BR-combined cold treatment at seed germination and bud burst stages. A high-resolution SNP genetic map, covering 1,145 bin markers with a distance of 3188.33 cM onto 12 chromosomes, was constructed using the GBS technique. A total of 73 QTLs were detected, of which 49 QTLs were identified under cold stress and 24 QTLs under BR-combined cold treatment. Among these, intervals of 30 QTLs were pairwise coincident under cold stress and BR-combined cold treatment, as well as different traits including SR and FLL, and PH and FLL, respectively. A total of 14 candidate genes related to cold tolerance or the BR signaling pathway, such as CBF/DREB (LOC_Os08g43200), bHLH (LOC_Os07g08440 and LOC_Os07g08440), WRKY (LOC_Os06g30860), MYB (LOC_Os01g62410 and LOC_Os05g51160), and BRI1-associated receptor kinase 1 precursor (LOC_Os06g16300), were located. Among these, the transcript levels of 10 candidate genes were identified under cold stress and BR-combined cold treatment by qRT-PCR. These findings provided an important basis for further mining the genes related to cold tolerance or the BR signaling pathway and understanding the molecular mechanisms of cold tolerance in rice.

## Introduction

Rice (*Oryza sativa* L.) is one of the most important crops for food production, which grows in tropical, subtropical, and temperate regions worldwide (Cheng et al., 2007). Cold stress is one of the most severe environmental factors limiting the growth, development, and yield formation of rice. It affects almost all growth stages of rice, including germination stage, bud burst stage, seedling stage, tillering stage, booting stage, flowering stage, and grain filling stage (Fujino et al., 2004; Xu et al., 2015). The germination rate and post-germination early seedling growth are two important traits that directly contribute to seedling vigor (Fujino et al., 2008). Cold stress at the germination and bud burst stages is the major limitation due to high sensitivity to cold at these stages, especially for the direct-seeded rice. It impairs the seed germination and early seedling growth of rice, which in turn leads to uneven stand establishment of seedling, delay of panicle development, spikelet sterility, and subsequent yield loss (Satoh et al., 2016; Najeeb et al., 2020). Therefore, improving the cold tolerance of germination and bud burst stages is an important objective in rice cultivation and breeding.

Brassinosteroids (BR) are essential plant steroid hormones that play important roles in growth, development, and tolerance to stresses in plants. Many studies have reported that exogenous BR application can obviously improve cold tolerance in plants (Chung et al., 2014; Sharma et al., 2017; Manghwar et al., 2022). In *Arabidopsis thaliana*, loss-of-function mutations of *brassinosteroid insensitive 1* (*BRI1*) and *brassinazole resistant 1* (*BZR1*) and overexpression of *brassinosteroid insensitive 2* (*BIN2*) result in decreased cold tolerance, whereas overexpression *of BRI1* and *BZR1* enhances cold tolerance (Li et al., 2017; Manghwar et al., 2022). In addition, BR biosynthetic genes *BR6ox2*, *DWF4*, and *CPD* are rapidly downregulated under cold stress (Chung et al., 2014; Eremina et al., 2016). In rice, soaking with BR can effectively alleviate the damage of rice seeds under cold stress, and spraying with BR can also improve cold tolerance of rice seedlings (Hwang and Back, 2019; Wu et al., 2020; Sadura and Janeczko, 2021). However, few genes related to both cold tolerance and BR in rice have been reported to date. The regulatory relationship between cold tolerance and BR in rice remains undefined.

Cold tolerance is a very complex quantitative trait associated with a massive number of biochemical and physiological processes and environment factors, which is genetically controlled by multiple quantitative trait loci (QTLs) or genes (Zhang et al., 2014). QTL mapping is one of the most common methods to identify the QTLs and genes related to cold tolerance in plants. Many genomic regions on all 12 rice chromosomes have been reported to contain some QTLs related to cold tolerance at different development stages (Miura et al., 2001; Fujino et al., 2004, 2008; Yang et al., 2021). For instance, *Ctb1* is the first cloned QTL for cold tolerance in rice. *Ctb1* encodes a F-box protein, which improves cold tolerance by associating with a subunit of the E3 ubiquitin ligase (Skp1) at the booting stage of rice (Saito et al., 2010). CTB4a, a leucine-rich repeat receptor-like kinase, increases ATP synthase activity and ATP content by interacting with the β-subunit of ATP synthase, which enhances seed setting and improves yield under low-temperature stress conditions (Zhang et al., 2017). In addition, COLD1 functions as a GTPase-accelerating factor and regulates G-protein signaling under cold stress in rice. COLD1 interacts with regulator of GTPase-activating protein 1 (RGA1), rapidly activates inward current and Ca^2+^ concentration, and ultimately enhances cold tolerance at the seedling stage in japonica rice (Ma et al., 2015). However, these are not sufficient to mine the useful genes and reveal the molecular mechanisms of cold tolerance in rice. With the rapid development of high-throughput sequencing technologies, faster and simpler ways to obtain single-nucleotide polymorphism (SNP) molecular markers based on sequencing technologies are increasingly used to perform QTL mapping, such as genotyping-by-sequencing (GBS), bulked segregant analysis (BSA), and bulked segregant RNA-seq (BSR). The introduction of these technologies has accelerated the identification of millions of SNPs across the whole genome (Jiang et al., 2016; Mielczarek and Szyda, 2016). In particular, GBS is becoming popular for QTL mapping, genetic diversity, and genomic selection, which has been successfully applied in QTL mapping for cold tolerance, salinity tolerance, aluminum tolerance, rice blast resistance, pericarp color, and some agronomic trait in rice (Spindel et al., 2013; Arbelaez et al., 2015; De Leon et al., 2016).

Wild rice (*Oryza rufipogon* Griff) is the relative ancestor of the cultivated rice, which can be used as a donor of novel and favorable alleles for rice breeding (Nakagahra et al., 1997; Atwell et al., 2014; Zhang et al., 2020). QTL mapping has been extensively used to identify the novel loci from wild rice, and these loci are expected to be used for improving the agronomic traits of rice (Koseki et al., 2010; Mao et al., 2015). Dongxiang wild rice (DXWR) possesses an extremely high innate tolerance to low-temperature stress at the seedling, booting, and flowering stages, and its underground stem can tolerate −12.8°C (Li et al., 2010; Zhang et al., 2014). Some genes or QTLs related to cold tolerance have been isolated and characterized in DXWR (Liu et al., 2003; Tian et al., 2006; Li et al., 2010; Xiao et al., 2015; Deng et al., 2018; Liang et al., 2018, 2019; Bai et al., 2021). Therefore, DXWR is an ideal germplasm for improving the cold tolerance of rice by hybridization, backcrossing, or genetic transformation.

In this study, we constructed a backcross recombinant inbred line (BRIL) population of 140 individuals derived from a cross between the DXWR and a super rice SN265 with excellent agronomic traits. On this basis, we used the GBS technique to construct a high-resolution genome-wide SNP genetic map for identification of cold-tolerant QTLs and candidate genes under cold stress and BR-combined cold treatment at the germination and bud burst stages. This study has important reference value for the discovery of cold tolerance genes and understanding of the regulation relationship between cold tolerance and BR in rice.

## Materials and Methods

### Plant Materials

The recipient parent SN265, a super rice variety with excellent agronomic traits from north China, was crossed with the donor parent DXWR, and the F_1_ plants were backcrossed with SN265 four times to develop the BC_4_F_1_ generation. The resulting BC_4_F_1_ lines were selfed and advanced by using the single seed descent method to generate 140 BRIL individuals in the F_8_ generation (BC_4_F_8_).

### Phenotypic Characterization for Cold Tolerance

The evaluation of cold tolerance at germination and bud burst stages was conducted based on previous studies with minor changes (Kim et al., 1999; Qiao et al., 2004; Akhtamov et al., 2020; Najeeb et al., 2020; Pan et al., 2020). Seeds of each accession from the BRILs were placed in a drying oven at 50°C for 72 h to break dormancy, and surface-sterilized in 1% NaClO solution for 10 min, followed by washing three times in sterilized distilled water. Then, 50 seeds were soaked with sterilized distilled water (for cold treatment) and 0.1 μmol/L BR solution (for BR-combined cold treatment) in a petri dish (15 cm) at a constant temperature of 20°C for 24 h, respectively. The 50 pre-soaked seeds were stressed at 10°C for 10 days, and then moved to a greenhouse at 25°C for recovery. The germinated seeds of each accession were counted daily and defined as the low-temperature germination rate (LTG). The germination rate was calculated as follows: Germination rate (%) = (number of germinated seeds/total number of seeds) × 100%. Cold tolerance based on the LTG was graded on a scale of 1–9 as follows: 1, more than 90%; 3, 80–90%; 5, 50–79%; 7, 1–49%; and 9, 0%.

The survival rate (SR) is the percentage of rice seedlings survived from the germination stage to the early seedling stage under cold stress. The pre-soaked seeds were germinated in a growth chamber at a constant temperature of 30°C for 24 h. The germinated seeds with 5-mm coleoptiles were stressed at 10°C for 7 days and then moved to a greenhouse at 25°C for 10 days to allow early seedlings to resume normal growth. The SR was calculated as follows: Survival rate (%) = (number of survival seedlings/number of buds) × 100%. At least five pots of 30 plants were assessed for each line. Cold tolerance based on the SR was graded on a scale of 1–9 as follows: 1, more than 90%; 3, 70–90%; 5, 50–70%; 7, 10–49%; and 9, less than 10%.

Cold stress can directly influence the growth of coleoptile length, leaf length, and plant height at the bud burst stage. Therefore, phenotypic evaluation of cold tolerance at the bud burst stage is significant (Qiao et al., 2004; Najeeb et al., 2020; Pan et al., 2020). In the current study, cold tolerance was evaluated based on the phenotypic changes of plant height (PH) and first leaf length (FLL) at the bud burst stage. The PH and FLL of the early seedlings after recovering for 10 days were measured. The average of the three replicates of 10 seedlings for each treatment was analyzed. For cold treatment, the data obtained under normal temperature (25°C) were used as control. Cold tolerance scores based on the reduction rate of the PH and FLL were calculated as follows: reduction rate of PH and FLL = [(PH or FLL under normal temperature − PH or FLL under cold treatment)/PH or FLL under normal temperature] × 100%. For BR-combined cold treatment, both data obtained under normal temperature and cold treatment were used as controls, respectively. Cold tolerance scores depend on the reduction rate of PH and FLL = [(PH or FLL under normal temperature − PH or FLL under BR-combined cold treatment)/PH or FLL under normal temperature] × 100%, or [(PH or FLL under BR-combined cold treatment − PH or FLL under cold treatment)/PH or FLL under BR-combined cold treatment] × 100%, respectively.

All experiments for evaluating cold tolerance at the bud burst stage were repeated three times under the same conditions, and the average cold tolerance scores from the three replicates were used for QTL mapping analysis. R software 3.5.1 was used to analyze frequency distributions and correlations of four phenotypic traits under their respective two treatments.^1^ The “cor” function was used to calculate Pearson’s correlation coefficient between any two treatments. The “hist” function computes a histogram of the average phenotypic scores of each individual under each treatment. The significance between any pair of treatment was classified as 0, 0.001, 0.01, 0.05, 0.1, and 1, which were denoted as “^**^,” “^**^,” “*,” “.,” and “”.

### Genotyping and Single-Nucleotide Polymorphism Identification

Fresh young leaves were collected from 14-day-old seedlings of 140 BRILs along with their parental lines and subjected to DNA extraction using a DNA extraction kit (Aidlab, China). GBS technology was used to construct DNA fragment libraries from each BRILs and their parental lines. *Pst*I and *Msp*I (NEB) were used for digestion and T4 ligase (NEB) for ligation. The libraries were enriched by PCR amplification and sequenced using an Illumina HiSeq 4000 instrument (Huada Gene Technology Co. Ltd., Shenzhen, China). The data were analyzed by Tassel software (Glaubitz et al., 2014). The raw reads were then filtered and sorted according to indices, and the high-quality SNPs between parents were termed by alignment with Nipponbare reference genome.

### Linkage Map Construction and Quantitative Trait Loci Mapping

The genotypic data from each BRILs with filtered SNP markers were used to construct linkage map using QTL IciMapping sofware v4.163. Furthermore, QTLs were mapped using the inclusive composite interval mapping of additive (ICIM-ADD) method. QTLs were computed by using a permutation test involving 1,000 runs at a significance level of *p* = 0.05. After completion of the permutation test, a window size of 10 cM and a walk speed of 1 cM were set to start analysis of composite interval mapping. The threshold for the logarithm of odds (LOD) scores was set to 3.0. QTL nomenclature was followed by the method of McCouch (2008).

### Annotation and Validation of Candidate Genes

The QTL intervals were determined by the physical position of the SNP markers, flanking the respective QTLs. All candidate genes were predicted in the located QTL ranges based on the Rice Genome Annotation Project website.^2^ The 3-day-old early seedlings with seeds from SN 265, DXWR, and three excellent cold-tolerant BRILs, and three non-excellent cold-tolerant BRILs at normal temperature, 12-h under cold stress, and 12-h BR-combined cold treatment (mentioned before for SR) were subjected to total RNA extraction using an RNA extraction kit (Aidlab, China). Three biological replicates were collected, immediately frozen in liquid nitrogen, and then stored at −80°C until further analysis. The real-time PCR (qRT-PCR) was performed to identify the expression patterns of 10 candidate genes. The qRT-PCR was carried out using a q225 Real-Time PCR System (Monad, China) under the following conditions: 95°C for 5 min; 30 cycles of 95°C for 10 s, 60°C for 30 s, and 72°C for 2 min; and 1 cycle of 72°C for 10 min. Each sample was analyzed in three biological and three technical replicates. The relative expression levels were calculated using the ΔΔCt method (Livak and Schmittgen, 2002). The *actin* gene of rice (LOC_Os03g50885) was used as a reference gene. ΔCt was the difference between the Cts (threshold cycles) of the target gene and Cts of the reference gene. ΔΔCt was calculated by subtracting ΔCt of the treatment group from ΔCt of the normal group. Fold change was calculated using the following formula: Fold change = 2^–ΔΔ*Ct*^. All primer sequences are listed in Supplementary Table 1.

## Results

### Phenotypic Characterization Under Cold Stress and Brassinosteroid-Combined Cold Treatment

**FIGURE 1:**
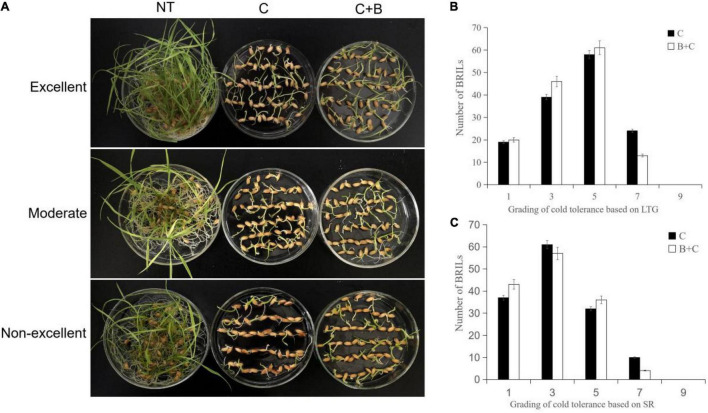
Phenotypic characterization under cold stress and BR-combined cold treatment at the germination stage in BRILs. **(A)** Phenotypes of the representative BRILs with excellent, moderate, and non-excellent cold tolerance. **(B)** Grading of cold tolerance based on LTG. **(C)** Grading of cold tolerance based on SR. LTG, low-temperature germination rate; SR, survival rate; NT, normal temperature; C, cold stress; C + B, BR-combined cold treatment.

**FIGURE 2:**
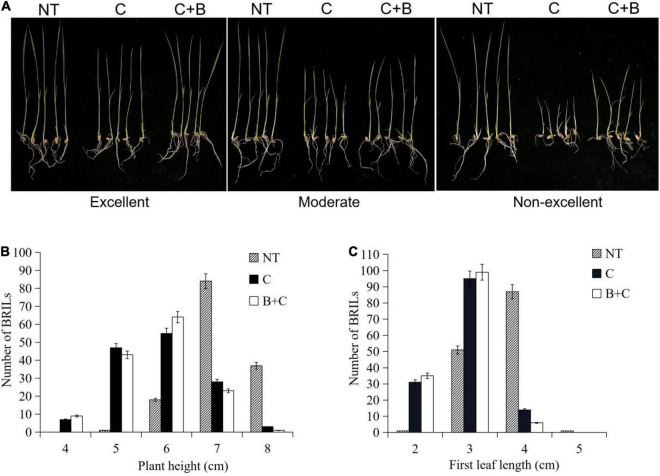
Phenotypic characterization under cold stress and BR-combined cold treatment at the bud burst stage in BRILs. **(A)** Phenotypes of the representative BRILs with excellent, moderate, and non-excellent cold tolerance. **(B)** Ranges of the plant height in BRILs. **(C)** Ranges of the first leaf length in BRILs. NT, normal temperature; C, cold stress; C + B, BR-combined cold treatment.

The BRIL population was evaluated under cold stress and BR-combined cold treatment at the germination and bud burst stages. The BRILs showed varying levels of cold tolerance and significantly contrasting responses in LTG, SR, PH, and FLL (Figures 1, 2). For LTG, grade 5 had the highest number of BRILs under cold stress, followed by grades 3, 7, and 1. Under BR-combined cold treatment, the number of BRILs in grades 3 and 5 increased, whereas the number of BRILs in grade 7 decreased (Figure 1B). For SR, grade 3 had the highest number of BRILs under cold stress, followed by grades 1, 5, and 7. After BR-combined cold treatment, the number of BRILs in grades 1 and 5 increased, whereas the number of BRILs in grade 7 decreased (Figure 1C). Furthermore, the phenotypic changes of PH and FLL were analyzed under cold stress and BR-combined cold treatment (Figure 2). Under normal temperature, the range of PH was 5−8 cm, and most of BRILs were distributed in the range of 7−8 cm, while the range of FLL was 2−4 cm, and most of BRILs were distributed in the range of 3−4 cm. After cold stress and BR-combined cold treatment, the PH and FLL were significantly reduced in most BRILs, with the range of 4−7 cm for PH and 2−4 cm for FLL, respectively. Compared to cold stress, the number of BRILs with PH of a 6-cm range and FLL of both 2-cm and 3-cm ranges increased under BR-combined cold treatment (Figures 2B,C). These results indicated that the continuous single-peak pattern distributions were observed for all four investigated traits, which are consistent with quantitative traits controlled by multiple genes. In addition, the treatment of BR could improve the ranges of LTG, SR, PH, and FLL in some BRILs according to the number of alterations of BRILs.

### Correlation Analysis of Low-Temperature Germination Rate, Survival Rate, Plant Height, and First Leaf Length

**FIGURE 3:**
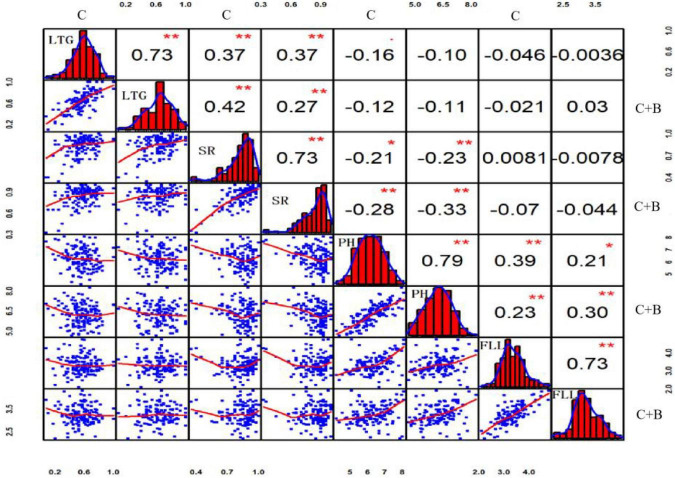
Correlation analysis of LTG, SR, PH, and FLL under cold stress and BR-combined cold treatment. Plots on the diagonal line show phenotypic distribution of each trait as indicated; values above diagonal line are Pearson’s correlation coefficients between traits; plots below diagonal line are scatter plots of compared traits. **P ≤ 0.01; *P ≤ 0.05. LTG, low-temperature germination rate; SR, survival rate; PH, plant height; FLL, first leaf length; C, cold stress; C + B, BR-combined cold treatment.

Pearson’s correlation analysis revealed that all four traits in the BRIL populations showed continuous and approximately normal distributions. Under cold stress, highly significant positive correlations were observed between LTG and SR (*r* = 0.37, *p* ≤ 0.01), and between PH and FLL (*r* = 0.39, *p* ≤ 0.01), respectively. By contrast, there was a significant negative correlation between SR and PH (*r* = −0.21, *p* ≤ 0.05). Under BR-combined cold treatment, the positive correlations between LTG and SR (*r* = 0.37, *p* ≤ 0.01), as well as between PH and FLL (*r* = 0.39, *p* ≤ 0.01), and a negative correlation between LTG and SR (*r* = 0.37, *p* ≤ 0.01) were shown similar to those under cold stress. Additionally, under both cold stress and BR-combined cold treatment, all of LTG, SR, PH, and FLL showed highly significant positive correlations with *r* = 0.73 (*p* ≤ 0.01), *r* = 0.73 (*p* ≤ 0.01), *r* = 0.79 (*p* ≤ 0.01), and *r* = 0.73 (*p* ≤ 0.01), respectively (Figure 3).

### Genetic Linkage Map of the Recombination Bin Markers

In total, 64.48 Gb of high-quality sequence data were obtained from 42.8 M raw reads *via* the GBS approach, and 96.19% of those reads were mapped to the Nipponbare reference genome. The ratio of Q20 for BRILs was above 90%, and the guanine–cytosine (GC) content was 43.55%; thus, the quality of the data met requirements for further analysis. Finally, a total of 10,836 unfiltered SNPs were validated for the determination of recombinant events. After filtration followed by the ABH plugin, a total of 1,145 recombination bin markers were obtained to construct a recombination map for the BRIL population.

A genetic linkage map was developed by mapping the 1,145 bin marker to the 12 rice chromosomes. The 12 rice linkage groups varied in the number of markers and marker density. The total genetic distance of linkage map was 3188.33 cM, with the individual linkage groups ranging from 186.74 to 346.46 cM in length. The highest number of markers was 117 on chromosome 4, and the lowest number was 77 on chromosome 7. The averaged genetic distance between markers was 0.3 cM, and the interval of genetic distance among markers ranged from 1.9 to 146.3 cM (Table 1).

**TABLE 1:** Summary of genetic linkage map characteristics in BRILs.

Chr	Number of bin markers	Genetic distance (cM)	Ave. genetic distance between markers (cM)	Ave. interval (cM)	Interval range (cM)
1	104	268.20	0.4	25.8	5.1–120.5
2	112	288.60	0.3	25.8	4.0–74.5
3	116	314.04	0.3	27.1	4.9–73.6
4	117	346.46	0.3	29.6	7.5–88.7
5	81	229.41	0.4	28.3	3.6–127.8
6	100	293.94	0.4	29.4	1.9–98.6
7	77	186.74	0.4	24.3	4.0–75.6
8	91	271.54	0.5	29.8	6.3–146.3
9	79	227.08	0.3	28.7	6.2–121.3
10	85	230.99	0.3	27.2	5.4–112.6
11	105	303.46	0.3	28.9	2.0–126.8
12	78	227.87	0.3	29.2	2.0–137.7
All	1,145	3188.33	0.3 (Ave.)	27.8 (Ave.)	1.9–146.3

*Chr, chromosome number; Ave, average.*

### Comprehensive Quantitative Trait Loci Mapping for Cold Tolerance in Backcross Recombinant Inbred Line Population

**FIGURE 4:**
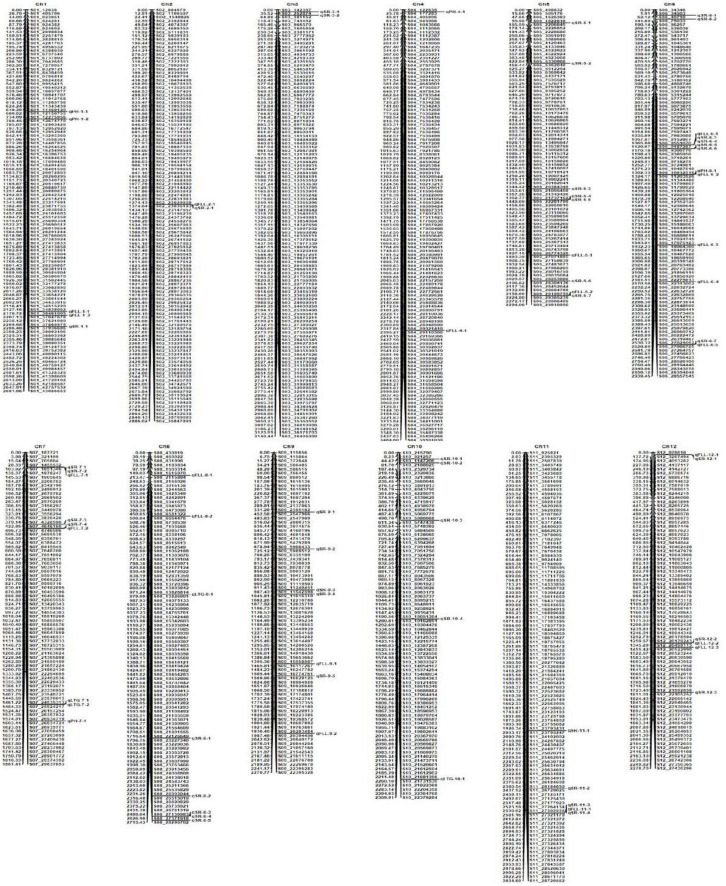
Molecular genetic map showing the positions of QTLs for four traits investigated under cold stress and BR-combined cold treatment.

The 1,145 SNP markers were used in QTL mapping of cold tolerance based on LTG, SR, PH, and FLL, respectively. An LOD threshold of 3.0 was used to identify QTLs for each trait using the CIM analysis. A total of 73 QTLs were detected, of which 49 QTLs were identified under cold stress and 24 QTLs under BR-combined cold treatment. The phenotypic variation explained (PVE) by these QTLs ranged from 0.52 to 14.69%, while the range of LOD threshold was 3.02−9.38 (Figure 4 and Table 2).

**TABLE 2:** QTL summary under different conditions for four traits.

Trait	Condition	QTL	Chr	Interval	LOD	PVE	Add
Germination rate	Cold	*qLTG7-1*	7	24535313–25924217	3.93	10.84	0.09
	Cold + BR	*qLTG7-2*	7	24535313–25924217	3.06	12.14	0.10
		*qLTG8-1*	8	13826814–13826901	3.24	6.40	–0.08
		*qLTG10-1*	10	21681306–21731530	3.48	4.74	–0.06
Seeding rate	Cold	*qSR1-1*	1	37869575–37936686	3.11	0.52	0.04
		*qSR2-1*	2	23029608–23036193	4.00	1.53	0.11
		*qSR3-1*	3	142357–252874	5.89	0.76	0.05
		*qSR5-1*	5	1622816–2206645	3.10	1.58	0.11
		*qSR5-5*	5	21737162–22281799	3.49	1.61	0.11
		*qSR5-6*	5	28253886–28304136	3.55	1.64	0.11
		*qSR5-7*	5	29195759–29365239	4.36	1.58	0.11
		*qSR6-1*	6	34503–49710	3.60	1.44	0.12
		*qSR6-2*	6	49710–75033	3.71	1.48	0.12
		*qSR6-3*	6	8551070–9187287	3.51	1.38	0.13
		*qSR6-5*	6	9187287–9300770	3.41	1.43	0.12
		*qSR6-7*	6	27021092–27066725	3.29	1.61	0.11
		*qSR7-2*	7	1455532–1851346	4.69	1.62	0.11
		*qSR7-3*	7	4328590–4646160	4.58	1.46	0.12
		*qSR7-4*	7	4646160–4746506	4.96	1.60	0.11
		*qSR8-1*	8	22126649–23227340	3.05	1.54	0.11
		*qSR8-2*	8	26093044–26515019	3.87	1.57	0.12
		*qSR8-3*	8	27136983–27371918	3.91	1.52	0.12
		*qSR9-1*	9	2395182–2547966	4.62	1.61	0.12
		*qSR9-2*	9	5908433–5989512	4.65	1.37	0.10
		*qSR9-3*	9	11240451–11542598	5.32	1.58	0.11
		*qSR9-4*	9	11542598–11616310	3.95	1.49	0.10
		*qSR9-5*	9	16774761–16829125	3.45	1.44	0.12
		*qSR10-3*	10	5080068–5747438	3.63	1.56	0.11
		*qSR10-4*	10	10051304–10162891	3.44	1.60	0.11
		*qSR11-1*	11	23793431–23795247	4.23	1.53	0.12
		*qSR11-2*	11	26184650–26729025	3.28	1.50	0.11
		*qSR11-4*	11	27302936–27321179	3.58	1.48	0.11
		*qSR12-1*	12	978610–3267494	4.14	1.55	0.12
		*qSR12-2*	12	20179243–20499113	3.99	1.56	0.12
		*qSR12-3*	12	21992858–22105252	3.60	1.55	0.11
	C + BR	*qSR3-2*	3	252874–381152	9.38	7.00	0.09
		*qSR5-2*	5	5330966–5362049	3.83	1.55	–0.04
		*qSR5-3*	5	20384349–21055459	3.33	2.76	–0.08
		*qSR5-4*	5	21737162–22281799	3.50	3.97	0.09
		*qSR6-4*	6	8551070–9187287	3.22	3.74	0.11
		*qSR6-6*	6	9187287–9300770	3.29	4.10	0.10
		*qSR7-1*	7	1455532–1851346	3.23	4.15	0.10
		*qSR8-4*	8	27136983–27371918	3.57	3.61	0.12
		*qSR8-5*	8	27371918–28296702	3.05	3.59	0.12
		*qSR10-1*	10	921257–1147206	4.85	4.20	0.09
		*qSR10-2*	10	1147206–2188025	3.31	3.91	0.09
		*qSR11-3*	11	27302936–27321179	3.59	4.11	0.10
Plant height	Cold	*qPH1-2*	1	12275956–12674770	3.89	11.71	0.05
	Cold + BR-N	*qPH1-1*	1	11469120–12150755	3.63	9.27	–0.05
		*qPH4-1*	4	122635–177283	4.73	8.94	–0.05
		*qPH7-1*	7	26934719–26934724	8.58	14.69	–0.07
	Cold + BR-C	*qPH6-1*	6	10912518–11203926	4.13	10.62	–0.05
First leaf length	Cold	*qFLL1-1*	1	34838052–36461005	3.34	1.37	–0.12
		*qFLL2-1*	2	23029608–23036193	3.35	1.39	–0.14
		*qFLL4-1*	4	29021419–29110306	3.92	1.35	–0.13
		*qFLL5-1*	5	26774329–27071895	3.14	1.39	–0.14
		*qFLL5-2*	5	29195759–29365239	3.66	1.41	–0.14
		*qFLL6-1*	6	8551070–9187287	3.26	1.34	–0.14
		*qFLL6-3*	6	17075123–18064360	3.14	1.38	–0.14
		*qFLL6-5*	6	27021092–27066725	3.65	1.41	–0.13
		*qFLL7-1*	7	1455532–1851346	3.02	1.36	–0.14
		*qFLL7-2*	7	4646160–4746506	3.40	1.34	–0.14
		*qFLL9-1*	9	15958987–16117267	3.02	1.34	–0.12
		*qFLL9-2*	9	20293484–20394456	3.34	1.27	–0.13
		*qFLL11-1*	11	27302936–27321179	3.22	1.30	–0.14
		*qFLL12-1*	12	978610–3267494	3.77	1.37	–0.15
		*qFLL12-2*	12	20179243–20499113	3.82	1.42	–0.15
		*qFLL12-3*	12	20499113–20522990	4.11	1.44	–0.15
	Cold + BR-N	*qFLL1-2*	1	36461005–37047678	3.12	11.12	–0.07
		*qFLL6-4*	6	22275613–22313002	4.12	8.92	0.06
	Cold + BR-C	*qFLL6-2*	6	10912518–11203926	4.49	7.66	–0.07
		*qFLL8-1*	8	1515970–2115884	4.68	9.81	–0.09
		*qFLL8-2*	8	6581322–6664476	3.28	7.33	–0.07

*Chr, chromosome number; LOD, logarithm of odds value; PVE, phenotypic variance explained; Add, additive effect; Cold, cold stress; Cold + BR, BR-combined cold treatment; Cold + BR-N, the data under normal temperature condition were used as the control; Cold + BR-C, the data under cold treatment condition were used as the control.*

A total of four QTLs related to LTG were localized on chromosomes 7, 8, and 10, respectively. Among them, *qLTG7-1* with 3.93 LOD and 10.84% PVE was localized on chromosome 7 under cold stress, while *qLTG7-2*, *qLTG8-1*, and *qLTG10-1*, with the LOD ranging from 3.06 to 3.48 and PVE ranging from 4.74 to 12.41%, were localized on chromosomes 7, 8, and 10 under BR-combined cold treatment, respectively. The interval of *qLTG7-1* localized under cold stress coincided with the interval of *qLTG7-2* localized under BR-combined cold treatment (Table 2; Supplementary Figure 1).

For SR, a total of 43 QTLs were identified on all chromosomes, except chromosome 4, with LOD ranging from 3.05 to 9.38 and PVE ranging from 0.52 to 7.0%, 31 of which were localized under cold stress and 12 were localized under BR-combined cold treatment, respectively. Intervals between some QTLs under both cold stress and BR-combined cold treatment were coincident, including *qSR5-4* and *qSR5-5*, *qSR6-4* and *qSR6-3*, *qSR6-6* and *qSR6-5*, *qSR7-1* and *qSR7-2*, *qSR8-4* and *qSR8-3*, and *qSR11-3* and *qSR11-4*, respectively (Table 2; Supplementary Figure 2).

Unlike the LTG and SR, the data analysis of PH and FLL for QTL mapping needed to set different treatments as the controls (see Section “Materials and Methods” for details). For PH, only one QTL (*qPH1-2*) was localized on chromosome 1 under cold stress. Under BR-combined cold treatment, three QTLs (*qPH1-1*, *qPH4-1*, and *qPH7-1*) were identified on chromosomes 1, 4, and 7 with the control of PH under the normal-temperature condition, while one QTL (*qPH6-1*) was localized on chromosome 6 with the control of PH under cold stress. The LOD values of these QTLs ranged from 3.63 to 8.58, and their PVE values ranged from 8.94 to 14.69% (Table 2; Supplementary Figure 3).

For FLL, a total of 21 QTLs with LOD ranging from 3.02 to 4.68 and PVE ranging from 1.27 to 11.12% were identified on all chromosomes, except chromosomes 3 and 10. Among these 21 QTLs, 16 were localized in cold stress and five were localized in BR-combined cold treatment. Under BR-combined cold treatment, two QTLs (qFLL1-2 and *qFLL6-4*) were identified on chromosomes 1 and 6 using PH under normal-temperature conditions as control, while three QTLs (*qFLL6-2*, *qFLL8-1*, and *qFLL8-2*) were localized on chromosomes 6 and 8 with PH under cold stress as control (Table 2; Supplementary Figure 4).

Interestingly, intervals between some QTLs related to different traits were also coincident including *qSR2-1* and *qFLL2-1*, *qSR5-7* and *qFLL5-2*, *qSR6-4* and *qFLL6-1*, *qSR6-7* and *qFLL6-5*, *qSR7-2* and *qFLL7-1*, *qSR7-4* and *qFLL7-2*, *qSR11-3* and *qFLL11-1*, and *qFLL6-2* and *qPH6-1*, respectively.

### Identification of Candidate Genes

**FIGURE 5:**
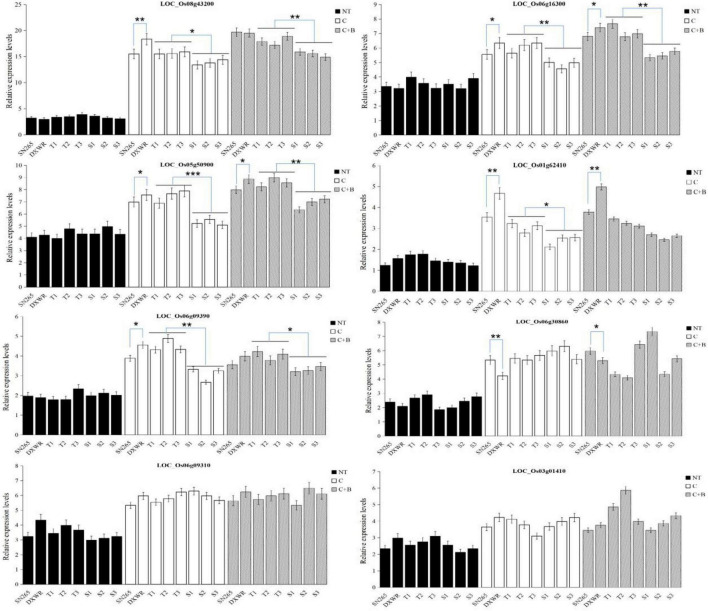
Expression patterns of the candidate genes. Biological triplicates were averaged and statistically analyzed *via* Student’s *t*-test (*P < 0.05; **P < 0.01; ***textitP < 0.001). NT, normal temperature; C, cold stress; C + B, BR-combined cold treatment.

According to the Rice Genome Annotation Project, the coincident QTL regions and QTLs with a higher LOD score (more than 5.0) were chosen to identify candidate genes using flanking markers. A total of 121 genes were located in the QTL regions mentioned previously, of which 60 were annotated with known functions, while the remaining 61 genes were identified as expressed proteins, hypothetical proteins, transposon, and retrotransposon proteins (Supplementary Table 2). Among these annotated candidate genes, 14 genes were possibly associated with cold tolerance or the BR signaling pathway based on the previously reported studies (Table 3). These genes included some transcription factors such as C-repeat-binding factor/DRE-binding factor (CBF/DREB, LOC_Os08g43200), basic helix–loop–helix (bHLH) transcription factor (LOC_Os07g08440 and LOC_Os05g50900), AP2 domain-containing proteins (LOC_Os06g44750 and LOC_Os06g09390), WRKY (LOC_Os06g30860), and MYB (LOC_Os01g62410 and LOC_Os05g51160), ethylene-responsive transcription factor (ERF, LOC_Os05g34730), and some other proteins including leucine-rich repeat (LRR) family protein (LOC_Os03g01410), heat shock factor-binding protein (LOC_Os06g16270), zinc finger-containing protein (LOC_Os06g09310), and extra-large G-protein-related (LOC_Os05g50910), and brassinosteroid insensitive 1 (BRI1)-associated receptor kinase 1 precursor (LOC_Os06g16300). Surprisingly, BRI1-associated receptor kinase 1 precursor, a protein associated with the BR signaling pathway, was just identified under BR-combined cold treatment. Furthermore, we validated the expression levels of 10 candidate genes under cold stress and BR-combined cold treatment using qRT-PCR. The results showed that the transcript levels of all genes, except LOC_Os05g50910 and LOC_Os05g34730, were significantly higher under both cold stress and BR-combined cold treatment than under normal temperature in all materials tested, while the transcript levels of LOC_Os06g16300, LOC_Os08g43200, and LOC_Os05g50900 under BR-combined cold treatment were slightly higher than those under cold stress. For two parents, LOC_Os05g50900, LOC_Os06g16300, and LOC_Os03g01410 showed higher expression levels in DXWR than in SN265 under cold stress and BR-combined cold treatment, whereas the opposite expression pattern was observed for LOC_Os06g30860 and LOC_Os06g09310. For the representative BRILs, LOC_Os05g50900, LOC_Os06g16300, LOC_Os08g43200, LOC_Os05g50900, and LOC_Os06g09390 showed higher transcript levels in three excellent BRILs than three non-excellent BRILs both under cold stress and BR-combined cold treatment (Figure 5; Supplementary Figure 5).

**TABLE 3:** Summary of the annotated candidate genes possibly associated with cold tolerance or the BR signaling pathway.

Condition	QTL	Chr	Locus name	Gene coordinates	Gene product
Cold/Cold + BR	qSR6-5/6	6	LOC_Os06g16300	9289219–9291362	BRASSINOSTEROID INSENSITIVE 1 Associated receptor kinase 1 precursor
Cold/Cold + BR	qSR8-3	8	LOC_Os08g43200	27321388–27320517	DREB dehydration-responsive element-binding protein
Cold	qFLL1-1	1	LOC_Os01g62410	36122778–36128487	MYB family transcription factor
Cold	qFLL5-2/qSR5-7	5	LOC_Os05g51160	29348631–29347196	MYB family transcription factor
Cold	qFLL6-3	6	LOC_Os06g30860	17917083–17915923	WRKY transcription factor 31
Cold + BR	qSR5-3	5	LOC_Os05g34730	20601854–20601261	Ethylene-responsive transcription factor ERF020
Cold	qSR7-3	7	LOC_Os07g08440	4338514–4342219	bHLH transcription factor
Cold	qFLL5-2/qSR5-7	5	LOC_Os05g50900	29211341–29208619	bHLH transcription factor
Cold/Cold + BR	qSR6-5/6	6	LOC_Os06g16270	9263601–9265449	Heat shock factor binding protein 2
Cold + BRs	qSR3-2	3	LOC_Os03g01410	294916–293668	Leucine rich repeat family protein
Cold	qFLL5-2/qSR5-7	5	LOC_Os05g50910	29213556–29219606	Extra-large G-protein-related
Cold	qFLL6-5/qSR6-7	6	LOC_Os06g44750	27025437–27029339	AP2 domain containing protein
Cold	qFLL7-2/qSR7-4	7	LOC_Os06g09390	4731330–4733911	AP2 domain containing protein
Cold	qFLL7-2/qSR7-4	7	LOC_Os06g09310	4678838–4680332	Zinc finger, C3HC4 type domain containing protein

*Chr, chromosome number; Cold, cold treatment; Cold + BR, BR-combined cold treatment.*

## Discussion

As a complex quantitative trait, cold tolerance in plants is controlled by various QTLs or genes. Thus, it is absolutely essential to mine more QTLs or genes to get a deeper understanding of molecular mechanisms for cold tolerance in plants (Zhang et al., 2014; Jha et al., 2017). Genetic diversity is the basis of the agronomic traits and stress resistance improvement in plants, and gene diversity is the most widely used index for measuring the level of genetic diversity. Wild species have high diversity of desirable genes for agronomic traits and resistance to stresses, and these allelic variations are important genetic resources for agronomic traits and stress resistance improvement (Koseki et al., 2010; Atwell et al., 2014). Common wild rice is the relative ancestor of the cultivated rice, which possesses abundant genetic diversity that can be used to improve agronomic traits and resistance to stress in cultivated rice. However, some gene resources might have been lost in cultivated rice after thousands of years of evolution and natural selection (Liu et al., 2003; Koseki et al., 2010; Liang et al., 2018, 2019; Zhang et al., 2020).

As previously mentioned, DXWR has a strong cold tolerance at the seedling, booting, and flowering stages, which is an ideal material for identifying and offering genes related to cold tolerance for rice improvement (Zhang et al., 2006). To date, many rice populations have been constructed using DXWR as the paternal parent, while many QTLs and candidate genes related to cold tolerance have been identified in these populations (Mao et al., 2015; Liang et al., 2018, 2019). In the current study, SN265, a super rice variety with excellent agronomic traits, was crossed with the donor parent DXWR to obtain F1 populations. To have a uniform background, the material was propagated by backcrossing the F1 populations with female parent SN265 for additional four times (BC_4_F_1_). Furthermore, the BC_4_F_1_ populations were selfed for multiple generations to generate an advanced BRIL population (BC_4_F_8_), and 140 BRIL individuals with abundant genetic diversity were chosen for cold tolerance evaluation and GBS sequencing. The backcrossing and selfing for multiple generations eliminated the background interference caused by different genetic bases, making the results of phenotypic evaluation and QTL mapping of cold tolerance more accurate and reliable.

Multiple studies have demonstrated that exogenous application of BR increased cold tolerance in plants. However, only several genes in BR biosynthesis and signaling pathways were reported to be involved in cold tolerance in Arabidopsis such as *BRI1*, *BZR1*, *BIN2*, *BR6ox2*, *DWF4*, and *CPD* (Chung et al., 2014; Eremina et al., 2016; Li et al., 2017; Sharma et al., 2017; Manghwar et al., 2022). In rice, few genes have been reported to be involved in both cold stress and BR biosynthesis or signaling pathways. In the current study, the exogenous application of BR improved the cold tolerance of some BRILs at germination and bud burst stages under cold stress, which provided a basis for identifying the QTLs related to cold stress and BR pathways.

The data of LTG and SR could be derived directly from the calculation of corresponding proportion under different treatments, and the analysis process does not need the controls (see Section “Materials and Methods” for details). Unlike LTG and SR, however, the measurements of PH and FLL for QTL mapping need to have a control so that the data could embody the changes under different treatment. Thus, the measurements of PH and FLL under cold stress set the data under normal-temperature condition as the control, whereas the measurements of PH and FLL under BR-combined cold treatment set the data under normal-temperature condition and cold stress as the controls, respectively. This was done to obtain more comprehensive QTLs (Kim et al., 1999; Qiao et al., 2004; Akhtamov et al., 2020; Najeeb et al., 2020; Pan et al., 2020).

In QTL mapping, intervals between some QTLs were coincident under different treatments (cold stress and BR-combined cold treatment), as well as different traits including SR and FLL, and PH and FLL, respectively. These coincident QTLs were probably more accurate. Furthermore, many candidate genes were identified by using the coincident QTL regions and those QTLs with higher LOD scores (more than 5.0). Among these genes, there were some transcription factors related to cold tolerance such as CBF/DREB (LOC_Os08g43200), bHLH (LOC_Os07g08440 and LOC_Os07g08440), WRKY (LOC_Os06g30860), MYB (LOC_Os01g62410 and LOC_Os05g51160), and ERF (LOC_Os05g34730). In particular, DREB/CBF, bHLH, and WRKY transcription factors have been reported to be involved in BR signaling pathways. Under cold conditions, BR directs a bHLH transcription factor CESTA (CES) to contribute to the constitutive expression of the CBF regulators that control the expression of cold responsive genes in Arabidopsis (Eremina et al., 2016). BZR1, a key transcription factor in the BR signaling pathway, acts upstream of CBF1 and CBF2 to directly improve cold tolerance in Arabidopsis. Moreover, BZR1 also regulates WKRY6 to modulate plant response to cold stress (Li et al., 2017). In the current study, qRT-PCR was applied to preliminarily characterize the transcript levels of all transcription factor genes under cold stress and BR-combined cold treatment. The transcript levels of these genes, except LOC_Os05g34730, were upregulated both under cold stress and BR-combined cold treatment. For the representative BRILs, LOC_Os05g50900 (bHLH), LOC_Os08g43200 (DREB/CBF), and LOC_Os05g50900 (bHLH) showed higher transcript levels in the excellent BRILs than in the non-excellent BRILs under cold stress and BR-combined cold treatment. In addition to the transcription factors, some genes related to cold tolerance were also identified such as LRR protein, heat shock factor-binding protein, and zinc finger-containing protein. Interestingly, a BRI1-associated receptor kinase 1 precursor (LOC_Os06g16300) was also identified under BR-combined cold stress. BRI1 is a leucine-rich repeat receptor-like kinase that functions as the cell surface receptor for BR, and loss-of-function mutation of *BRI1* resulted in decreased cold tolerance in Arabidopsis (Eremina et al., 2016; Xu et al., 2022). Although the aforementioned candidate genes have been reported to be involved in cold tolerance or BR signaling pathways, few genes related to both cold stress and BR pathways have been studied in rice. In the current study, we initially demonstrated that the transcript levels of LOC_Os06g16300 (BRI1-associated receptor kinase 1), LOC_Os08g43200 (DREB/CBF), and LOC_Os05g50900 (bHLH) under BR-combined cold treatment were higher than those under cold stress. Further studies are needed to demonstrate whether these genes are involved in cold tolerance and BR pathways in rice. In conclusion, many QTLs and candidate genes were identified by GBS sequencing and mapping analysis, which provided an important basis for further mining the genes related to cold tolerance or BR pathways and studying the molecular mechanisms regulating cold tolerance in rice.

## Data Availability Statement

The original contributions presented in the study are publicly available. This data can be found here: NCBI, PRJNA836720.

## Author Contributions

ML and MZ designed and supervised the research work. ZG, MZ, and ML constructed the materials. ZG, HW, and JY determined phenotypic data, mapping analysis, and qRT-PCR experiments. YC and WZ performed the candidate gene association. JH and ZX contributed to revising the manuscript. All authors contributed to the article and approved the submitted version.

## Conflict of Interest

The authors declare that the research was conducted in the absence of any commercial or financial relationships that could be construed as a potential conflict of interest.

## Publisher’s Note

All claims expressed in this article are solely those of the authors and do not necessarily represent those of their affiliated organizations, or those of the publisher, the editors and the reviewers. Any product that may be evaluated in this article, or claim that may be made by its manufacturer, is not guaranteed or endorsed by the publisher.

## Footnotes

1
www.R-project.org

2
http://rice.plantbiology.msu.edu
