# Supplementary material for: Quantitative Trait Loci Mapping Analysis for Cold Tolerance Under Cold Stress and Brassinosteroid-Combined Cold Treatment at Germination and Bud Burst Stages in Rice
Source: Front Plant Sci. 2022 Jul 18;13:938339. doi: 10.3389/fpls.2022.938339 (PMC9340073; doi:10.3389/fpls.2022.938339)
Supplement: Supplementary file 1 [file Data_Sheet_1.docx]

**
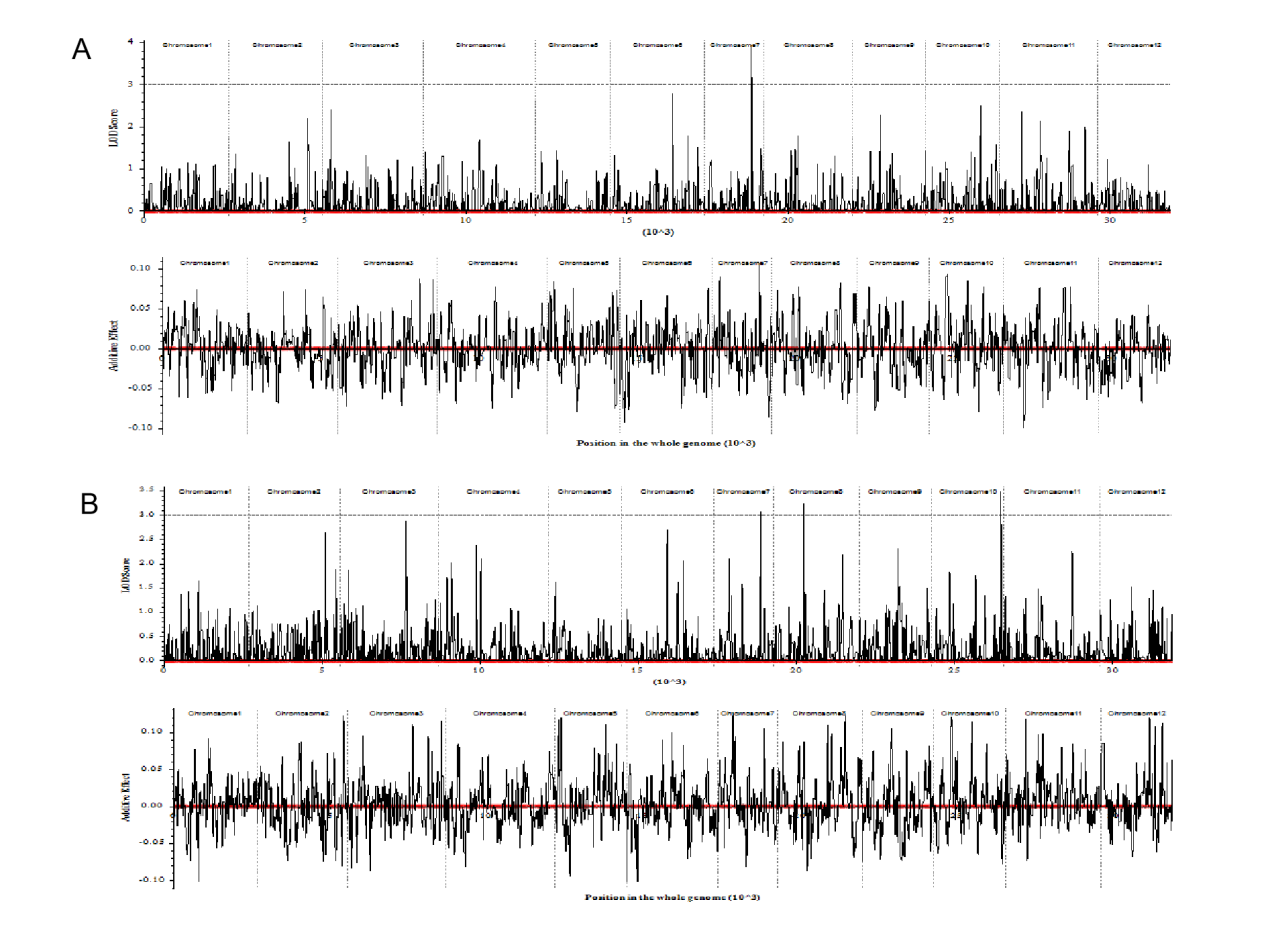
**

**Figure S1 |** QTL mapping based on low temperature germination rate (LTG). (**A**) Cold treatment. (**B**) BR combined cold treatment.

.

**
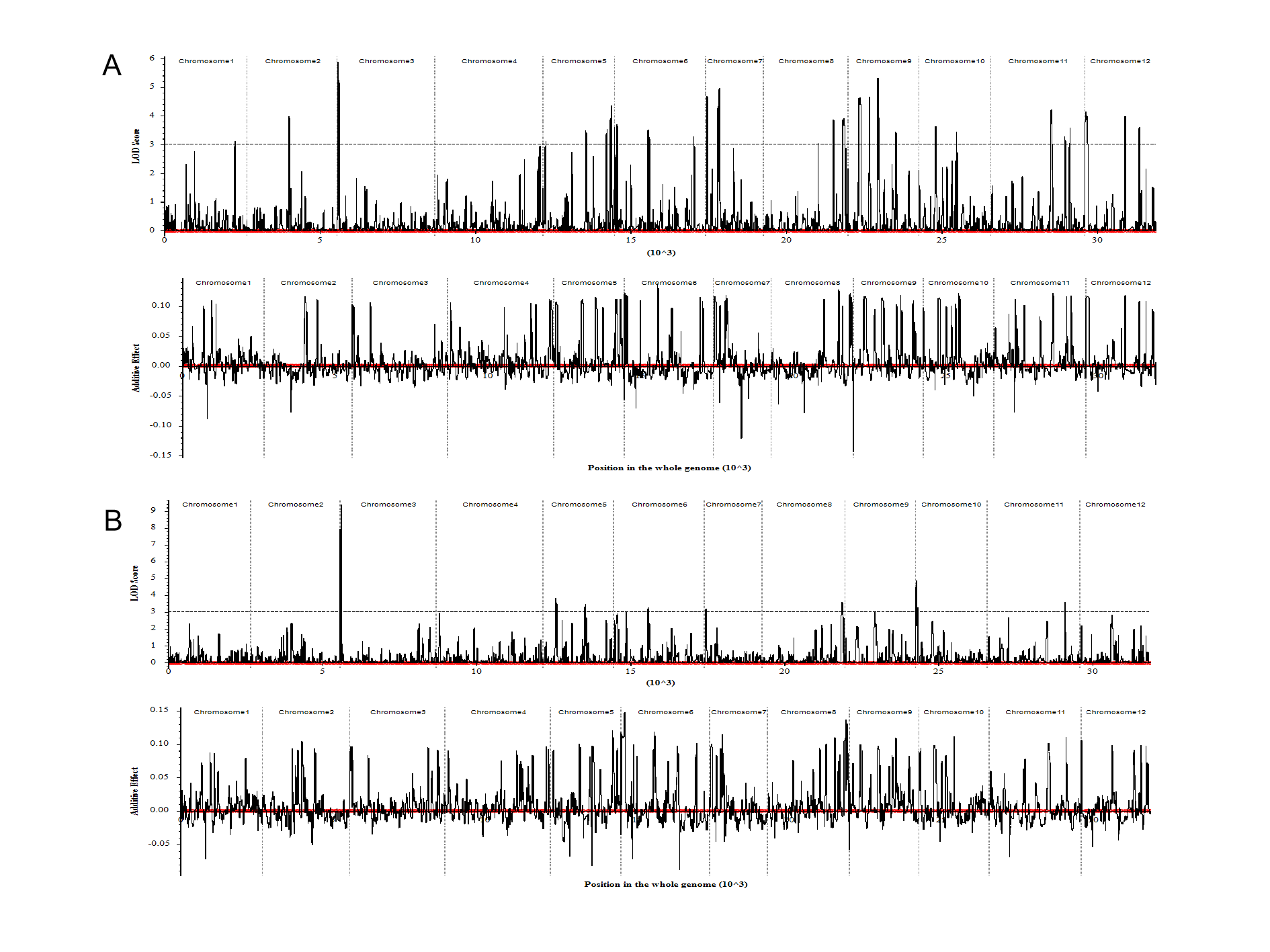
Figure S2 |** QTL mapping based on seedling rate (SR). (**A**) Cold treatment. (**B**) BR combined cold treatment.

**
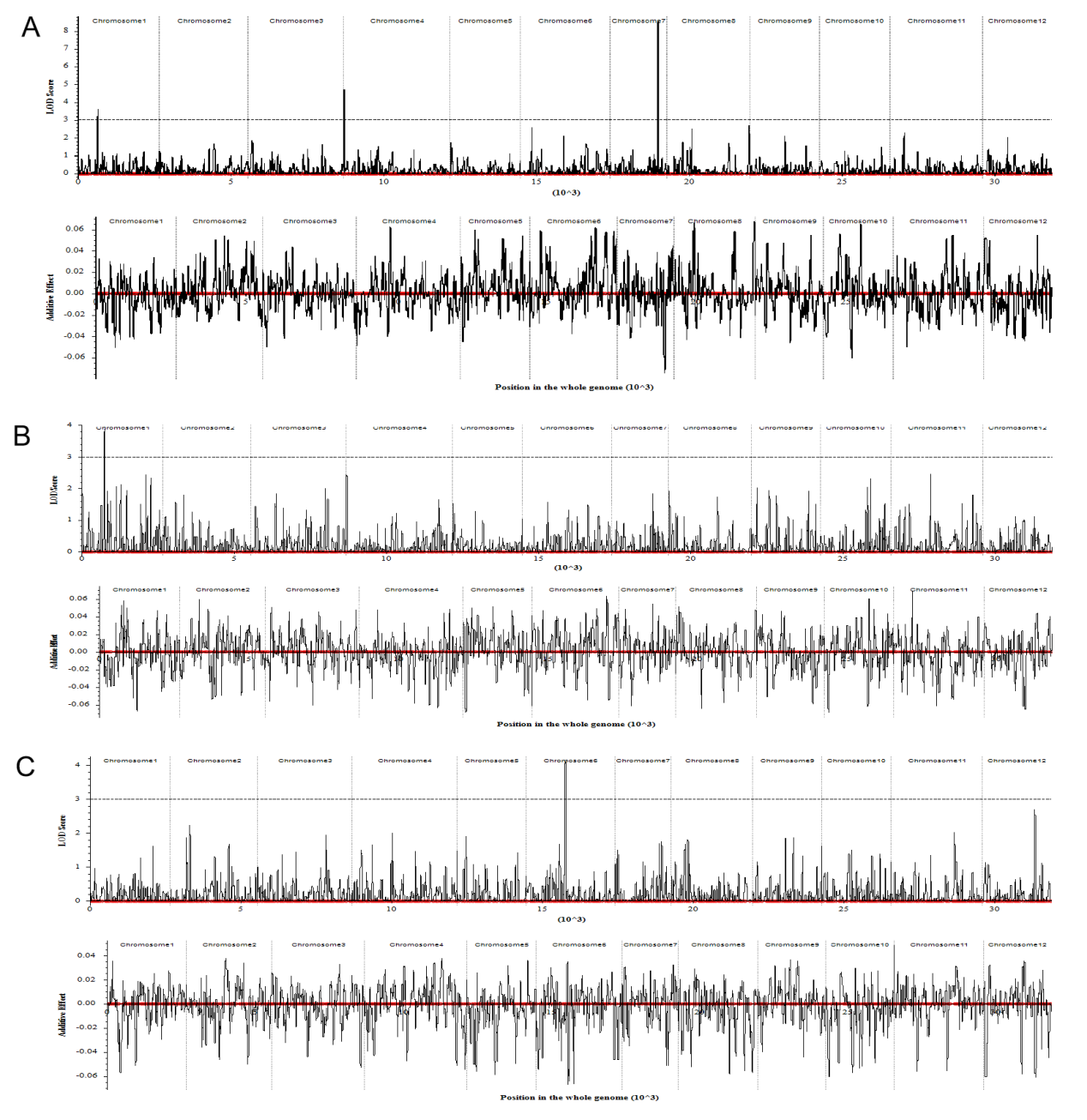
**

**Figure S3 |** QTL mapping based on plant height (PH). (**A**) Cold treatment. (**B**) BR combined cold treatment when normal temperature condition was used as the control. (**C**) BR combined cold treatment when cold treatment condition was used as the control. **
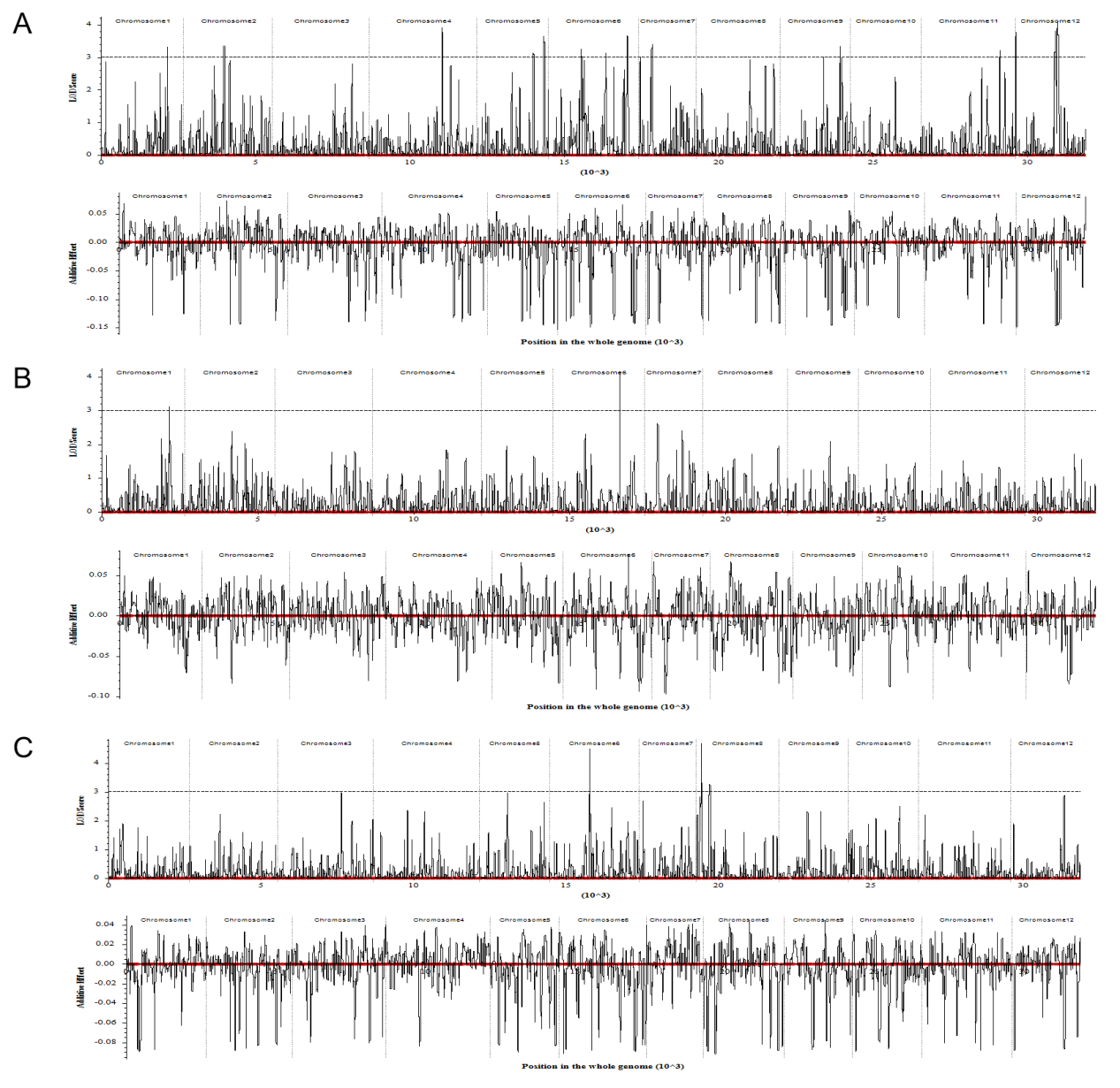
**

**Figure S4 |** QTL mapping based on first leaf length (FLL). (**A**) Cold treatment. (**B**) BR combined cold treatment when normal temperature condition was used as the control. (**C**) BR combined cold treatment when cold treatment condition was used as the control.

**
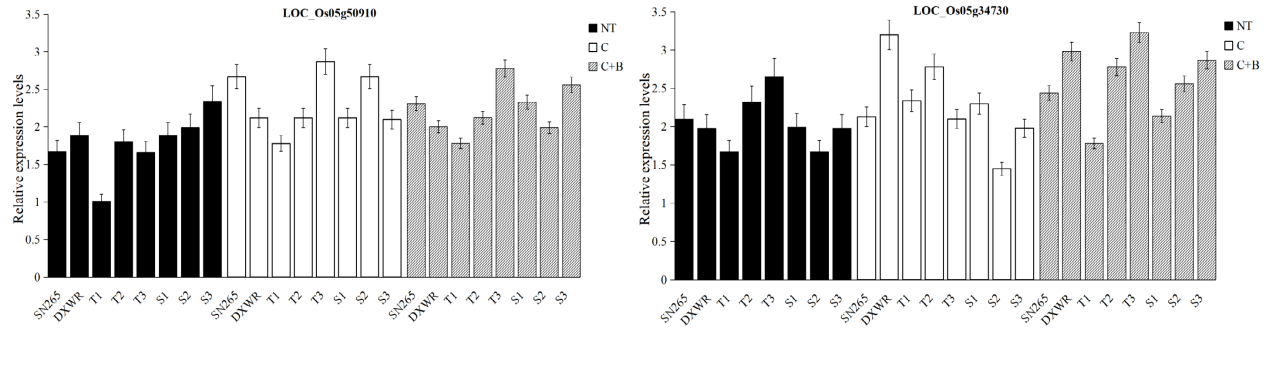
**

**Figure S5 |** Expression patterns of the candidate genes (LOC_Os05g50910 and LOC_Os05g34730). NT, normal temperature; C, Cold stress; C+B, BR combined cold treatment.

**Table S1|** Details of primers used for this study

| **Gene ID or Name** | **Forward primer (5’-3’)** | **Reverse primer (5’-3’)** |
| --- | --- | --- |
| LOC_Os06g16300 | GACTCCCTCGTCACTACCCT | CTTGCCAATCTCTGCTGGGA |
| LOC_Os08g43200 | TGTCACCCAACGACGACAAT | AGCTCGCGAAGTACAGATCG |
| LOC_Os01g62410 | GAAGTTCTTCCTCCGCCACA | GAGTGGCAGGATCGGATGAG |
| LOC_Os06g30860 | CAACAGGAGGTGCTTCAGGT | CCACCTCCTTTCTCGTCTGC |
| LOC_Os05g34730 | GCAAGAAAGCAAGGGACACG | CAATGAGCTCCTCACTCCCG |
| LOC_Os05g50900 | GTCGCGGACATAGAGGTGAG | TGCAGGATGGACATGTGCAT |
| LOC_Os03g01410 | CGGAAATGGCGCTCTTCATG | GAGATGCCCACCAGTATCGG |
| LOC_Os06g09390 | TTGTTCCTGCAATGAACGCG | TCAACACCTGTCATGGTCGG |
| LOC_Os05g50910 | TTCATGGCCAGAGAGCTGTG | GCTCCGATGAGACTGATGGG |
| LOC_Os06g09310 | CACCAACGTCCTCTTCTGGG | CGATGACGAGGCTCTCCTTC |
| LOC_Os03g50885(*actin*) | TCGAGCATGGTATCGTCAGC | GGATGGCGACGTACATAGCA |

**Table S2|** Summary of all candidate genes

| **Condition** | **QTL** | **Chr** | **Accession** | **Gene interval** | **Description** |
| --- | --- | --- | --- | --- | --- |
| Cold/Cold+BR | qSR8-3/4 | 8 | LOC_Os08g43200 | 27321388– 27320517 | dehydration-responsive  element-binding protein |
| Cold | qFLL1-1 | 1 | LOC_Os01g62410 | 36122778– 36128487 | MYB family transcription factor |
| Cold | qFLL6-3 | 6 | LOC_Os06g30860 | 17917083 - 17915923 | WRKY transcription factor 31 |
| Cold+BR | qSR5-3 | 5 | LOC_Os05g34730 | 20601854 - 20601261 | ethylene-responsive transcription factor ERF020 |
| Cold | qSR7-3 | 7 | LOC_Os07g08440 | 4338514 - 4342219 | BHLH transcription factor |
| Cold | qLTG7-1 | 7 | LOC_Os07g41370 | 11475315..11475545 | MADS-box family gene with  MIKCc type-box |
| Cold+BR | qLTG7-2 | 7 | LOC_Os07g41370 | 24788476 - 24793884 | MADS-box family gene with  MIKCc type-box |
| Cold/Cold+BR | qSR6-5/6 | 6 | LOC_Os06g16300 | 9289219 - 9291362 | associated receptor kinase 1  precursor, putative, expressed |
| Cold/Cold+BR | qSR6-5/6 | 6 | LOC_Os06g16290 | 9283140 - 9285250 | ribosomal protein L7Ae,  putative, expressed |
| Cold/Cold+BR | qSR6-5/6 | 6 | LOC_Os06g16200 | 9221326 - 9223650 | proteins of unknown function  domain containing protein,  expressed |
| Cold/Cold+BR | qSR6-5/6 | 6 | LOC_Os06g16270 | 9263601 - 9265449 | heat shock factor binding protein 2, putative, expressed |
| Cold/Cold+BR | qSR6-5/6 | 6 | LOC_Os06g16260 | 9262251 - 9256958 | OsSigP5 - Putative Type I  Signal Peptidase homologue;  employs a putative Ser/His  catalytic dyad, expressed |
| Cold/Cold+BR | qSR11-4/3 | 11 | LOC_Os11g45130 | 27318942 - 27324163 | pollen signalling protein with  adenylyl cyclase activity,  putative, expressed |
| Cold | qSR3-1 | 3 | LOC_Os03g01260 | 180206 - 179154 | expansin precursor, putative,  expressed |
| Cold | qSR3-1 | 3 | LOC_Os03g01300 | 208330 - 211839 | Protease  inhibitor/seed storage/LTP  family protein precursor,  expressed |
| Cold | qSR3-1 | 3 | LOC_Os03g01222 | 144603 - 147769 | formyl transferase, putative,  expressed |
| Cold | qSR3-1 | 3 | LOC_Os03g01270 | 189333 - 191297 | expansin precursor, putative, expressed |
| Cold | qSR3-1 | 3 | LOC_Os03g01320 | 219977 - 221070 | LTPL116 - Protease  inhibitor/seed storage/LTP  family protein precursor,  expressed |
| Cold | qSR3-1 | 3 | [LOC_Os03g01230](http://rice.uga.edu/cgi-bin/ORF_infopage.cgi?orf=LOC_Os03g01230.1) | 148183 - 149057 | DNA-directed RNA polymerase  II subunit RPB9, putative,  expressed |
| Cold | qSR3-1 | 3 | [LOC_Os03g01330](http://rice.uga.edu/cgi-bin/ORF_infopage.cgi?orf=LOC_Os03g01330.1) | 236750 - 235794 | sodium/potassium/calcium  exchanger 1, putative, expressed |
| Cold | qSR3-1 | 3 | LOC_Os03g01240 | 152358 - 170125 | CCR4-Not complex component,  Not1domain containing protein,  expressed |
| Cold | qSR3-1 | 3 | LOC_Os03g01290 | 202527 - 205750 | peptide transporter PTR2,  putative, expressed |
| Cold+BR | qSR3-2 | 3 | [LOC_Os03g01365](http://rice.uga.edu/cgi-bin/ORF_infopage.cgi?orf=LOC_Os03g01365.1) | 278705 - 277183 | protein phosphatase-2c,  putative, expressed |
| Cold+BR | qSR3-2 | 3 | LOC_Os03g01365 | 278705 - 277183 | protein phosphatase-2c,  putative, expressed |
| Cold+BR | qSR3-2 | 3 | LOC_Os03g0141 | 294916 - 293668 | Leucine Rich Repeat family  protein, expressed |
| Cold+BR | qSR3-2 | 3 | [LOC_Os03g01530](http://rice.uga.edu/cgi-bin/ORF_infopage.cgi?orf=LOC_Os03g01530.1) | 339986 - 341796 | tubulin/FtsZ domain containing  protein, putative, expressed |
| Cold+BR | qSR3-2 | 3 | LOC_Os03g0154 | 347004 - 344307 | DNA binding protein, putative,  expressed |
| Cold+BR | qSR3-2 | 3 | LOC_Os03g01590 | 374690 - 370151 | putative, expressed |
| Cold | qFLL2-1/qSR2-1 | 2 | LOC_Os05g50890 | 29200198 - 29205717 | Probable indole-3-acetic acid-amido synthetase, expressed |
| Cold | qFLL5-2/qSR5-7 | 5 | LOC_Os05g50900 | 29211341 - 29208619 | helix-loop-helix DNA-binding  protein, putative, expressed |
| Cold | qFLL5-2/qSR5-7 | 5 | LOC_Os05g50910 | 29213556 - 29219606 | extra-large G-protein-related,  putative, expressed |
| Cold | qFLL5-2/qSR5-7 | 5 | LOC_Os05g50920 | 29221404 - 29224479 | transmembrane amino acid  transporter protein, putative,  expressed |
| Cold | qFLL5-2/qSR5-7 | 5 | LOC_Os05g50930 | 29225038 - 29227623 | RNA polymerase sigma factor,  putative, expressed |
| Cold | qFLL5-2/qSR5-7 | 5 | LOC_Os05g50950 | 29236686 - 29240151 | pentatricopeptide repeat protein  PPR986-12, putative, expressed |
| Cold | qFLL5-2/qSR5-7 | 5 | LOC_Os05g50960 | 29242468 - 29240182 | polygalacturonase, putative,  expressed |
| Cold | qFLL5-2/qSR5-7 | 5 | LOC_Os05g50970 | 29249088 - 29244022 | protein phosphatase 2C,  putative, expressed |
| Cold | qFLL5-2/qSR5-7 | 5 | LOC_Os05g50980 | 29251632 - 29255944 | SET domain containing protein,  expressed |
| Cold | qFLL5-2/qSR5-7 | 5 | LOC_Os05g50990 | 29262204 - 29257803 | TTL3, putative, expressed |
| Cold | qFLL5-2/qSR5-7 | 5 | LOC_Os05g51020 | 29286852 - 29284540 | leaf senescence related protein,  putative, expressed |
| Cold | qFLL5-2/qSR5-7 | 5 | LOC_Os05g51040 | 29292436 - 29293646 | tesmin/TSO1-like CXC domain  containing protein, expressed |
| Cold | qFLL5-2/qSR5-7 | 5 | LOC_Os05g51050 | 29297308 - 29293825 | acyl-protein thioesterase,  putative, expressed |
| Cold | qFLL5-2/qSR5-7 | 5 | LOC_Os05g51060 | \| 29304267 - 29300928 \| \| --- \| | DNA ligase, putative, expressed |
| Cold | qFLL5-2/qSR5-7 | 5 | LOC_Os05g51070 | 29307315 - 29311681 | inactive receptor kinase  At2g26730 precursor, putative,  expressed |
| Cold | qFLL5-2/qSR5-7 | 5 | LOC_Os05g51090 | 29314868 - 29312987 | nodulin MtN3 family  protein,putative, expressed |
| Cold | qFLL5-2/qSR5-7 | 5 | [LOC_Os05g51100](http://rice.uga.edu/cgi-bin/ORF_infopage.cgi?orf=LOC_Os05g51100.1) | 29316599 - 29317087 | F-box domain containing protein, expressed |
| Cold | qFLL5-2/qSR5-7 | 5 | LOC_Os05g51130 | 29333774 - 29331744 | mitochondrial chaperone BCS1,  putative, expressed |
| Cold | qFLL5-2/qSR5-7 | 5 | [LOC_Os05g51150](http://rice.uga.edu/cgi-bin/ORF_infopage.cgi?orf=LOC_Os05g51150.1) | 29341241 - 29345481 | RNA polymerase sigma factor,  putative, expressed |
| Cold | qFLL5-2/qSR5-7 | 5 | LOC_Os05g51160 | 29348631 - 29347196 | Myb transcription factor,  putative, expressed |
| Cold | qFLL5-2/qSR5-7 | 5 | LOC_Os05g51180 | 29355973 - 29359209 | hyaluronan/mRNA binding  family domain containing  protein, expressed |
| Cold | qFLL5-2/qSR5-7 | 5 | LOC_Os05g51190 | 29364139 - 29359930 | protein kinase family protein, putative, expressed |
| Cold | qFLL6-5/qSR6-7 | 6 | LOC_Os06g44750 | 27025437 - 27029339 | AP2 domain containing protein,  expressed |
| Cold | qFLL7-2/qSR7-4 | 7 | LOC_Os06g09240 | 4647954 - 4649523 | anthocyanidin  3-O-glucosyltransferase,  putative, expressed |
| Cold | qFLL7-2/qSR7-4 | 7 | LOC_Os06g09340 | 4700489 - 4703227 | pectinesterase, putative,  expressed |
| Cold | qFLL7-2/qSR7-4 | 7 | LOC_Os06g09390 | 4731330 - 4733911 | AP2 domain containing protein,  expressed |
| Cold | qFLL7-2/qSR7-4 | 7 | [LOC_Os06g09290](http://rice.uga.edu/cgi-bin/ORF_infopage.cgi?orf=LOC_Os06g09290.2) | 4667486 - 4670980 | 26S protease regulatory subunit  7, putative, expressed |
| Cold | qFLL7-2/qSR7-4 | 7 | [LOC_Os06g09330](http://rice.uga.edu/cgi-bin/ORF_infopage.cgi?orf=LOC_Os06g09330.1) | 4695316 - 4690734 | ubiquitin-conjugating enzyme,  putative, expressed |
| Cold | qFLL7-2/qSR7-4 | 7 | LOC_Os06g09270 | 4655652 - 4653457 | hypro1, putative, expressed |
| Cold | qFLL7-2/qSR7-4 | 7 | LOC_Os06g09310 | 4678838 - 4680332 | zinc finger, C3HC4 type domain  containing protein, expressed |
| Cold | qFLL7-2/qSR7-4 | 7 | [LOC_Os06g09280](http://rice.uga.edu/cgi-bin/ORF_infopage.cgi?orf=LOC_Os06g09280.1) | 4659377 - 4667061 | ATP-dependent RNA helicase,  putative, expressed |
| Cold | qFLL7-2/qSR7-4 | 7 | LOC_Os06g09370 | 4715358 - 4709743 | PTF1, putative, expressed |
| Cold/Cold+BR | qFLL11-1/qSR11-3 | 11 | LOC_Os11g45130 | 27318942 - 27324163 | pollen signalling protein with  adenylyl cyclase activity, putative, expressed |
| Cold/Cold+BR | qSR11-4/3 | 11 | LOC_Os11g45120 | 27308209 - 27309827 | conserved hypothetical protein |
| Cold | qSR3-1 | 3 | LOC_Os03g01216 | 139453 - 144463 | conserved hypothetical protein |
| Cold+BR | qSR3-2 | 3 | LOC_Os03g01500 | 325982 - 324860 | hypothetical protein |
| Cold+BR | qSR3-2 | 3 | [LOC_Os03g01500](http://rice.uga.edu/cgi-bin/ORF_infopage.cgi?orf=LOC_Os03g01500.1) | 325982 - 324860 | hypothetical protein |
| Cold | qFLL7-2/qSR7-4 | 7 | LOC_Os06g09300 | 4673672 - 4672872 | hypothetical protein |
| Cold/Cold+BR | qFLL11-1/qSR11-3 | 11 | LOC_Os11g45120 | 27308209 - 27309827 | conserved hypothetical protein |
| Cold/Cold+BR | qSR6-5/6 | 6 | LOC_Os06g16220 | 9234086 - 9237323 | transposon protein, putative,  unclassified, expressed |
| Cold/Cold+BR | qSR6-5/6 | 6 | LOC_Os06g16230 | 9244110 - 9237953 | transposon protein, putative,  unclassified, expressed |
| Cold+BR | qSR3-2 | 3 | LOC_Os03g01390 | 286798 - 284933 | transposon protein, putative,  unclassified, expressed |
| Cold | qFLL6-5/qSR6-7 | 6 | LOC_Os06g44800 | 27067208 - 27063980 | transposon protein, putative,  Ac/Ds sub-class, expressed |
| Cold/Cold+BR | qSR6-5/6 | 6 | LOC_Os06g16180 | 9215597 - 9215328 | retrotransposon protein,  putative, unclassified |
| Cold/Cold+BR | qSR6-5/6 | 6 | LOC_Os06g16310 | 9294370 - 9297555 | retrotransposon protein,  putative, unclassified, expressed |
| Cold/Cold+BR | qSR6-5/6 | 6 | LOC_Os06g16320 | 9304351 - 9298391 | retrotransposon protein,  putative, unclassified, expressed |
| Cold+BR | qSR3-2 | 3 | [LOC_Os03g01380](http://rice.uga.edu/cgi-bin/ORF_infopage.cgi?orf=LOC_Os03g01380.1) | 281563 - 283893 | retrotransposon protein,  putative, unclassified, expressed |
| Cold+BR | qSR3-2 | 3 | [LOC_Os03g01400](http://rice.uga.edu/cgi-bin/ORF_infopage.cgi?orf=LOC_Os03g01400.1) | 289958 - 288792 | retrotransposon protein,  putative, unclassified, expressed |
| Cold | qFLL5-2/qSR5-7 | 5 | [LOC_Os05g51010](http://rice.uga.edu/cgi-bin/ORF_infopage.cgi?orf=LOC_Os05g51010.1) | 29280523 - 29276615 | retrotransposon protein,  putative, Ty1-copia subclass,  expressed |
| Cold | qFLL5-2/qSR5-7 | 5 | LOC_Os05g51170 | 29354229 - 29350007 | retrotransposon protein,  putative, Ty1-copia subclass,  expressed |
| Cold | qFLL5-2/qSR5-7 | 5 | LOC_Os05g51010 | 29280523 - 29276615 | retrotransposon protein,  putative, Ty1-copia subclass,  expressed |
| Cold | qFLL5-2/qSR5-7 | 5 | LOC_Os05g51170 | 29354229 - 29350007 | retrotransposon protein,  putative, Ty1-copia  subclass, expressed |
| Cold/Cold+BR | qSR6-5/6 | 6 | [LOC_Os06g16160](http://rice.uga.edu/cgi-bin/ORF_infopage.cgi?orf=LOC_Os06g16160.1) | 9206466 - 9202757 | - |
| Cold/Cold+BR | qSR6-5/6 | 6 | LOC_Os06g16250 | 9253724 - 9255540 | - |
| Cold/Cold+BR | qSR6-5/6 | 6 | LOC_Os06g16280 | 9280989 - 9267068 | - |
| Cold/Cold+BR | qSR6-5/6 | 6 | LOC_Os06g16150 | 9197709 - 9196353 | - |
| Cold/Cold+BR | qSR6-5/6 | 6 | [LOC_Os06g16170](http://rice.uga.edu/cgi-bin/ORF_infopage.cgi?orf=LOC_Os06g16170.1) | 9209513 - 9207750 | - |
| Cold/Cold+BR | qSR6-5/6 | 6 | LOC_Os06g16190 | 9219660 - 9219869 | - |
| Cold/Cold+BR | qSR6-5/6 | 6 | LOC_Os06g16210 | 9231628 - 9229868 | - |
| Cold/Cold+BR | qSR6-5/6 | 6 | LOC_Os06g16240 | 9246577 - 9248376 | - |
| Cold | qSR3-1 | 3 | [LOC_Os03g01250](http://rice.uga.edu/cgi-bin/ORF_infopage.cgi?orf=LOC_Os03g01250.1) | 175129 - 169854 | - |
| Cold | qSR3-1 | 3 | [LOC_Os03g01280](http://rice.uga.edu/cgi-bin/ORF_infopage.cgi?orf=LOC_Os03g01280.1) | 196580 - 197599 | - |
| Cold+BR | qSR3-2 | 3 | LOC_Os03g01350 | 262977 - 266238 | - |
| Cold+BR | qSR3-2 | 3 | LOC_Os03g01360 | 268599 - 274808 | - |
| Cold+BR | qSR3-2 | 3 | LOC_Os03g01420 | 297898 - 295311 | - |
| Cold+BR | qSR3-2 | 3 | [LOC_Os03g01436](http://rice.uga.edu/cgi-bin/ORF_infopage.cgi?orf=LOC_Os03g01436.1) | 304170 - 305342 | - |
| Cold+BR | qSR3-2 | 3 | [LOC_Os03g01442](http://rice.uga.edu/cgi-bin/ORF_infopage.cgi?orf=LOC_Os03g01442.1) | 308488 - 305842 | - |
| Cold+BR | qSR3-2 | 3 | [LOC_Os03g01450](http://rice.uga.edu/cgi-bin/ORF_infopage.cgi?orf=LOC_Os03g01450.1) | 310402 - 309073 | - |
| Cold+BR | qSR3-2 | 3 | LOC_Os03g01460 | 311887 - 312675 | - |
| Cold+BR | qSR3-2 | 3 | LOC_Os03g01470 | 313806 - 315335 | - |
| Cold+BR | qSR3-2 | 3 | LOC_Os03g01430 | 317555 - 316096 | - |
| Cold+BR | qSR3-2 | 3 | [LOC_Os03g01490](http://rice.uga.edu/cgi-bin/ORF_infopage.cgi?orf=LOC_Os03g01490.1) | 322414 - 323643 | - |
| Cold+BR | qSR3-2 | 3 | LOC_Os03g01520 | 338711 - 335493 | - |
| Cold+BR | qSR3-2 | 3 | LOC_Os03g01550 | 352329 - 348339 | - |
| Cold+BR | qSR3-2 | 3 | [LOC_Os03g01560](http://rice.uga.edu/cgi-bin/ORF_infopage.cgi?orf=LOC_Os03g01560.1) | 355863 - 356213 | - |
| Cold+BR | qSR3-2 | 3 | [LOC_Os03g01570](http://rice.uga.edu/cgi-bin/ORF_infopage.cgi?orf=LOC_Os03g01570.1) | 359721 - 356719 | - |
| Cold+BR | qSR3-2 | 3 | [LOC_Os03g01580](http://rice.uga.edu/cgi-bin/ORF_infopage.cgi?orf=LOC_Os03g01580.2) | 367356 - 361703 | - |
| Cold+BR-W | qPH7-1 | 7 | LOC_Os07g45120 | 26934311 - 26937417 | - |
| Cold | qFLL5-2/qSR5-7 | 5 | LOC_Os05g50940 | 29228254 - 29229250 | - |
| Cold | qFLL5-2/qSR5-7 | 5 | [LOC_Os05g51000](http://rice.uga.edu/cgi-bin/ORF_infopage.cgi?orf=LOC_Os05g51000.1) | 29274798 - 29271238 | - |
| Cold | qFLL5-2/qSR5-7 | 5 | LOC_Os05g51030 | 29287647 - 29289703 | - |
| Cold | qFLL5-2/qSR5-7 | 5 | LOC_Os05g51080 | 29312656 - 29311991 | - |
| Cold | qFLL5-2/qSR5-7 | 5 | LOC_Os05g51110 | 29324891 - 29317524 | - |
| Cold | qFLL5-2/qSR5-7 | 5 | LOC_Os05g51119 | 29327061 - 29330145 | - |
| Cold | qFLL5-2/qSR5-7 | 5 | LOC_Os05g51140 | 29336108 - 29340508 | - |
| Cold | qFLL6-5/qSR6-7 | 6 | LOC_Os06g44760 | 27043124 - 27045828 | - |
| Cold | qFLL6-5/qSR6-7 | 6 | LOC_Os06g44770 | 27047926 - 27046712 | - |
| Cold | qFLL6-5/qSR6-7 | 6 | LOC_Os06g44780 | 27054106 - 27054779 | - |
| Cold | qFLL6-5/qSR6-7 | 6 | LOC_Os06g44790 | 27062657 - 27059070 | - |
| Cold | qFLL7-2/qSR7-4 | 7 | LOC_Os06g09250 | 4650153 - 4649581 | - |
| Cold | qFLL7-2/qSR7-4 | 7 | LOC_Os06g09260 | 4650896 - 4651519 | - |
| Cold | qFLL7-2/qSR7-4 | 7 | LOC_Os06g09350 | 4704882 - 4703899 | - |
| Cold | qFLL7-2/qSR7-4 | 7 | LOC_Os06g09380 | 4721427 - 4720600 | - |
